# Fuzzy-Based Dynamic Time Slot Allocation for Wireless Body Area Networks

**DOI:** 10.3390/s19092112

**Published:** 2019-05-07

**Authors:** Sangeetha Pushpan, Bhanumathi Velusamy

**Affiliations:** Department of Electronics and Communication Engineering, Anna University Regional Campus, Coimbatore 600 025, India; vbhanu_02@yahoo.com

**Keywords:** wireless body area networks, slot allocation, fuzzy, fog computing, healthcare, medium access

## Abstract

With the advancement in networking, information and communication technologies, wireless body area networks (WBANs) are becoming more popular in the field of medical and non-medical applications. Real-time patient monitoring applications generate periodic data in a short time period. In the case of life-critical applications, the data may be bursty. Hence the system needs a reliable energy efficient communication technique which has a limited delay. In such cases the fixed time slot assignment in medium access control standards results in low system performance. This paper deals with a dynamic time slot allocation scheme in a fog-assisted network for a real-time remote patient monitoring system. Fog computing is an extended version of the cloud computing paradigm, which is suitable for reliable, delay-sensitive life-critical applications. In addition, to enhance the performance of the network, an energy-efficient minimum cost parent selection algorithm has been proposed for routing data packets. The dynamic time slot allocation uses fuzzy logic with input variables as energy ratio, buffer ratio, and packet arrival rate. Dynamic slot allocation eliminates the time slot wastage, excess delay in the network and attributes a high level of reliability to the network with maximum channel utilization. The efficacy of the proposed scheme is proved in terms of packet delivery ratio, average end to end delay, and average energy consumption when compared with the conventional IEEE 802.15.4 standard and the tele-medicine protocol.

## 1. Introduction

Wireless body area networks (WBANs) is growing rapidly due to the recent advancements in the fields of electronics, intelligent sensors, and wireless communication technologies [1]. WBAN is a type of wireless sensor network [2] that requires a number of nodes to be worn on the body or implanted within the human body to collect the health vital signs. It can also be considered as a subclass of wireless sensor networks (WSNs) [3,4] with certain specific characteristics that make the research challenges more exigent [5]. The sensors collect data periodically or aperiodically and route them through different body controller nodes using various routing protocols. A geographic delay tolerant network (DTN) routing protocol is presented in [6], with the primary objective to improve the routing efficiency and reduce the chance of selecting inappropriate nodes for routing. Greedy forwarding, perimeter forwarding, and DTN forwarding modes are used for efficient routing towards the destination. The paper [7] explained the need for programming frameworks and middlewares for collaborative body sensor networks (CBSNs) due to the complex system requirements of CBSNs, unlike star topology-based body sensor networks (BSNs). The paper presented a novel collaborative-signal processing in node environment (C-SPINE) framework for CBSNs. It was developed as an extension of Signal Processing In Node Environment (SPINE) middleware that was discussed in [8]. SPINE was designed to meet the high-level software abstraction and hardware constraints in single BSNs.

The medical applications of WBAN include daily monitoring of human health vital signs and detection of chronic diseases such that the treatment benefits the patient at an early stage. The challenging tasks in patient monitoring systems are high throughput, limited delay, and less energy consumption. However, the existing protocols are less efficient to meet these challenges. It means the body sensors must be low power devices with guaranteed reliability since battery replacement or recharging is difficult. Hence, this necessitates energy efficient and reliable MAC protocol. The IEEE 802.15.4 MAC is a low power standard with minimum delay requirements that is widely used in WBANs. However, it is less efficient in terms of delay, throughput, and energy consumption for periodic patient monitoring applications. In case of an unexpected event or life-critical applications, the channel and bandwidth utilization are poor for this standard. The two major channel access methods used in WBANs are carrier sense multiple access with collision avoidance (CSMA/CA) and time division multiple access (TDMA). In CSMA/CA, the nodes compete for the channel before the data transmission. In TDMA, each node can transmit during its assigned time slot. The total time is divided into equal time slots which are organized as superframes. In a superframe, a node can transmit data within a time slot.

In the IEEE 802.15.4 standard, the aontention access period (CAP) uses CSMA/CA and contention free period (CFP) uses guaranteed time slot (GTS) allocation based on TDMA [9]. There are some shortcomings in the case of life-critical WBAN applications with equal time slots. The first one is the bandwidth under-utilization, where nodes use only a small portion of the assigned slot. This leads to slot wastage which represents an empty slot in the CFP. The second is the limited GTS slots. This affects the medical scenarios where a number of life-critical events occur simultaneously. In this standard, only seven GTS slots are available which cannot accommodate the multiple emergency events in time. Another limitation is the fixed time slots in the superframe which fails during urgent situations.

With the introduction of the internet of things (IoT) and cloud computing [10,11] paradigms to the field of medical services, a number of healthcare systems have been developed in order to provide fast and reliable treatment to patients. It also includes the sharing of medical information among the medical institutions, family members and the related personnel [12]. IoT-based health applications are not sufficient for pervasive monitoring, which requires additional analysis and decision-making capabilities. In order to overcome this shortcoming, IoT enabled cloud-assisted monitoring services came up. However, these also suffered due to discontinuities in the network connectivity [13]. Hence, an extended version of cloud computing, called fog computing [14] or fogging, is used now, in which computations can be done in any node, called fog node or nodes at the edge of personal area network (PAN).

In a similar manner to cloud computing, fog nodes are also prone to failures. However, the impact of failure is less and easier to handle for fogging in comparison with the cloud [15]. Cloud failure affects the entire hospital, whereas fog failure is restrained to a smaller area such as a hospital ward or a block. In short, fog computing can overcome the limitations of cloud computing, including high bandwidth constraints, dependency on network infrastructure, unpredictable time of response from the cloud for emergency cases, and so on. The fogging has a shorter response time, as the data processing is carried out at the edge of the network and close to the source along with securing the data within the network.

**Figure 1:**
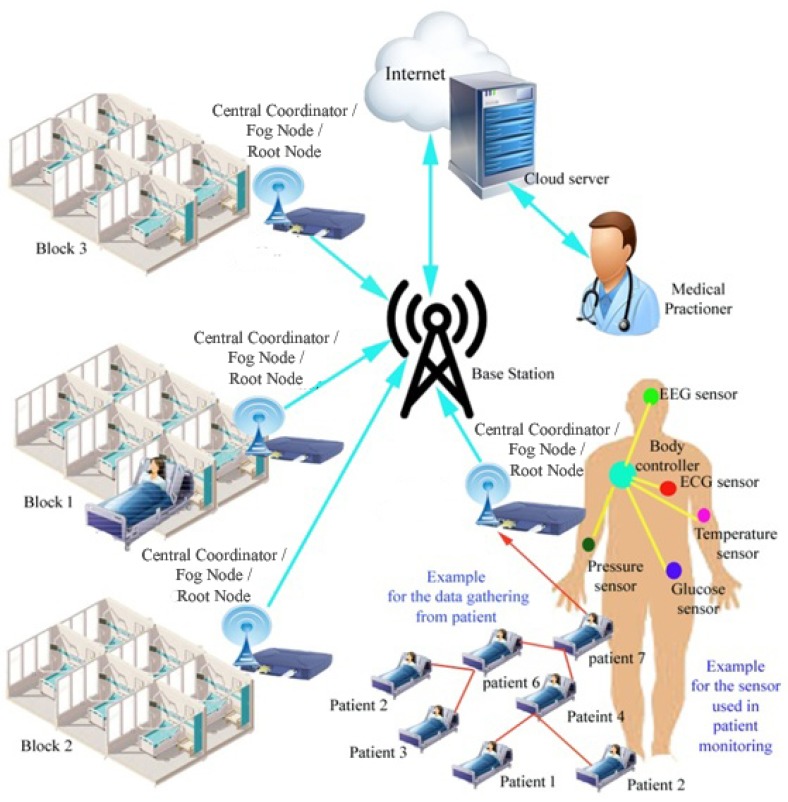
Fog-assisted architecture for in-hospital health management.

Figure 1 shows an example of an in-hospital block-wise health monitoring setup that utilizes the fog computing concept [16]. Each block has a number of patients and a central coordinator. In this method, the central coordinator (battery-operated node) acts as an edge computing device or a fog node. The central coordinator classifies the sensor signal into urgency, semi-urgency, and normal data by using simple mathematical models and a threshold value, and makes the decision accordingly (i.e., whether to immediately send the data to the base station or not). Then, the central coordinator directly sends the data to the base station (BS). The health monitoring system usually consists of a number of sensor nodes worn on the patient’s body, such as an electrocardiogram (ECG) sensor, electroencephalogram (EEG) sensor, temperature sensor, pressure sensor, glucose sensor, and a body controller. These sensor devices collect data from the body and send them to the body controller, which is placed at any appropriate position on the body. The body controller aggregates the collected information and sends it towards a central coordinator through the tree-based routes.

The advantage of fog computing is that the central coordinator or the fog node sends only the valid information and drops unnecessary sensor information, thereby simplifying the complexity of data storage and computation. It also makes the decision quickly. For example, in speech analysis for Parkinson’s disease, the audio recordings are not merely forwarded. Instead, the analysis of the recordings is done locally, and only the necessary metrics are transmitted [15]. Hence, the fog-architecture minimizes the delay which makes it suitable for various medical applications.

In this paper, a fog-based architecture and dynamic slot allocation are considered to address the discussed challenges of WBANs. The performance of an in-hospital patient monitoring system is enhanced by using a QoS efficient next hop selection algorithm and a fuzzy-based dynamic slot allocation scheme. The proposed methods are designed without modifying the superframe structure of the IEEE 802.15.4 MAC standard. The main contributions of the paper are:

- A fog-based WBAN for a real-time patient monitoring system which consists of a sensor layer, body controller layer, and a central coordinator layer.
- Minimum cost parent selection (MCPS) algorithm for best parent selection and a link cost function for efficient routing. The best parent node for the tree formation is selected by comparing the link cost function, number of hops, and the distance between the nodes.
- Dynamic time slot (DTS) allocation technique based on fuzzy logic that can enhance the packet delivery and reduce the end-to-end delay. The time slot to each node is allocated dynamically based on the parameters such as available energy in a node, buffer availability and the packet arrival rate.

The remaining paper is structured as follows: Section 2 summarizes some of the existing medium access control (MAC) layer protocols. Section 3 explains the system model for in-hospital health management application. Section 4 illustrates the tree formation and the cost function evaluation for energy efficient routing. Section 5 includes the design of an energy efficient dynamic time-slot allocation for each sensor node. Section 6 presents the performance results and analysis of the MCPS and DTS algorithm. Finally, Section 7 concludes the paper.

## 2. Related Works

The commonly utilized mechanisms in the MAC layer are time division multiple access (TDMA) and carrier sense multiple access with collision avoidance (CSMA/CA). Both of these mechanisms have their own advantages and disadvantages [17] in terms of power consumption, bandwidth utilization, network dynamics, synchronization, etc. A number of MAC layer protocols have been proposed, which combine the advantages of CSMA/CA and TDMA techniques in order to meet different demands such as reduction in the collisions, energy consumption and enhancement of the network reliability. In [18], MAC protocols with a quality of service (QoS) control scheme has been developed; however, they are not optimized for handling emergency data in medical applications. For an energy-efficient network, the MAC protocols in WBAN use duty-cycling mechanisms, which serves as an effective solution for over-hearing and idle listening problems. The beacon mode in IEEE 802.15.4 provides a better duty-cycling mechanism for using the available energy resources efficiently [19]. At the same time, this standard also faces several challenges such as unfair channel access, extended back off periods, and lack of dynamic adaptive capabilities. Hence, these issues result in inferior performance of WBAN in cases where the application demands less delay, accurate throughput, energy utilization and reliability at a specific time.

A new MAC protocol has been proposed in [20], which reduces the energy consumption of the guard band and extends the lifetime of the WBAN system. It uses a self-adaptive guard band in each time slot in order to reduce the energy consumption of the network. An enhanced packet scheduling algorithm (EPSA) is proposed in [21] to minimize the slot wastage and to allocate a greater number of waiting nodes in the available time slots. Initially, the vacant time slots are identified and divided into equal time slots based on the number of waiting nodes. Hence, they can transmit the data with a minimum delay in the given time frame. This scheme is based on the availability of the vacant time slots. The iQueue-MAC is a hybrid protocol [22] of CSMA/TDMA specifically designed for variable or bursty traffic. During low traffic it uses CSMA and when traffic increases it changes to TDMA mechanism. It uses a piggybacked indicator with a request for time slots. It allocates slots when a queue is detected. An energy preserving MAC protocol was derived in [23], called as Q-learning medium access control (QL-MAC) protocol with its aim to converge to a low energy state. It eliminated the need of a predetermined system model to solve the minimization problem in WSNs. It is also designed as a self-adaptive protocol against topological and other external changes.

In [24], a time slot allocation is modeled and proposed a time slot allocation scheme based on a utility function. The function is designed based on sensor priority, sampling rate and available energy of the node. The main objective is to maximize the data transmission of each node in the network. A priority-based adaptive MAC(PA-MAC) protocol [25] is derived for WBANs which dynamically allocates time slots to the nodes based on the traffic priority. There are separate channels for a beacon and data. A priority-guaranteed CSMA/CA is used to prioritize the data. Based on the traffic priority, the PA-MAC dynamically allocates the time slots. In [26] a Traffic Class Prioritization based CSMA/CA (TCP-CSMA/CA) is proposed for prioritized channel access in intra-WBAN. The aim is to reduce delay, minimize packet loss, and enhance network lifetime and throughput. The traffic is categorized into different classes and assigned backoff period range to each class.

To overcome the first-come-first-served (FCFS) guaranteed time slot (GTS) policy of IEEE 802.1.5.4 based network, an adaptive and real-time GTS allocation scheme (ART-GAS) is proposed in [27]. Here, the bandwidth utilization of IEEE 802.15.4 MAC for time-critical applications was improved. It used a two-stage approach, where the first stage dynamically assigned the priorities of all devices. In the second stage, the GTS was allocated to the nodes according to the assigned priorities. An analysis of the GTS allocation mechanism was done in [28] for time-critical applications based on the IEEE 802.15.4 standard. A Markov chain was considered to model the GTS allocation for designing various efficient GTS allocation schemes. In [29], real-time applications with periodic data are guaranteed with a reduced the packet drop rate. This algorithm can be used for only GTS allocation and it does not have any effect on the data packets in the contention access period (CAP).

The tele-medicine protocol (TMP) defined in [30] is a MAC protocol suitable for patient monitoring applications which need limited delay and reasonable reliability. The duty cycle is varied with respect to three parameters like delay-reliability factor, traffic load, and superframe duration. The protocol is designed based on three computation methods like network traffic estimation, channel access, and collision probabilities and delay-reliability factor. It shows the efficacy in terms of delay, reliability and efficient energy consumption.

A number of routing protocols are proposed and studied for routing the packets from source node to the sink node based on the tree structure. In [31], a routing protocol for low-power and lossy (RPL) Network is introduced where two routers along with interconnecting devices are restrained. It is based on IPv6 protocol which supports multipoint-to-point and point-to-point traffic within the lossy networks. It discusses the topologies like destination-oriented directed acyclic graphs (DODAGs), their upward and downward routes, security mechanisms, and fault management. A velocity energy-efficient and link-aware cluster-tree (VELCT) is proposed in [32] which provides reliable data collection scheme in sensor networks. Cluster head location is utilized to construct the data collection tree (DCT). It minimizes the energy consumption of the cluster head with less frequent cluster formation. It is well suitable for mobility based sensor networks. In [33], a cluster based routing protocol is introduced to extend the network lifetime of sensor networks. The energy of all nodes is balanced to prolong the lifetime of the network. It utilized a spanning tree to send heterogeneous data to the base station. A tree-based routing protocol (TBRP) is discussed in [34] for mobile sensor networks. It enhanced the node’s lifetime by considering different energy levels in the tree. Here, the lowest energy level consumes high energy and highest level consumes less energy. Whenever a node attained a critical level of energy, it saves the energy by moving into the next energy level.

The tree formation and routing of packets are influenced by the link reliability and the co-existence issues in the network. For context-aware WBAN, it has to coexist with a number of wireless networks. The paper [35] discussed the characteristics of the physical layer in a smart environment. The experiment characterized on-body and off-body channels. The author had come up with some concerns for physical layer protocol design. In [36], the co-channel interference in WBAN is addressed where it has to co-exist within smart environments operating in the same frequency band. It also discussed the fading characteristics of mobile WBAN. The measurements for inter-body interference between two WBANs are also explained. The reliability, fault-tolerant, and interference mitigation schemes are presented in [37]. The term reliability is expressed in terms of quality of the link and the efficiency of the communication. A detailed explanation about different types of interference and coexistence is also included. A decentralized time-synchronized channel swapping (DT-SCS) protocol is presented in [38] to overcome the shortcomings of time-synchronized channel hopping (TSCH) in ad hoc networks. These protocols were designed for collision-free and interference avoiding communications. The TSCH and its variants need centralized coordination technique for time-frequency slotting in networks. It resulted in slow convergence to the steady state during mobility. Hence, Dt-SCS was introduced with a decentralized concept based on the coupling of distributed synchronization and desynchronization mechanisms.

All the existing aforementioned approaches mainly concentrated on any one of the QoS aspects at a time, whereas a combined set of QoS parameter optimization is necessary for WBAN medical applications. Additionally, most of the MAC protocols based on the IEEE 802.15.4 standard concentrated on any one of the MAC aspects for the protocol design. Most of the schemes used data traffic and traffic priority for the analysis. Also, the developed protocols attained their objectives by adjusting the CAP/CFP in the superframe structure, which has its own limitations in terms of bandwidth and number of devices used. The comparative survey of different routing protocols for WBAN medical applications is summarized in [39].

## 3. System Model

### 3.1. Network Model

An in-hospital real-time healthcare patient monitoring network is assumed to evaluate the performance of the proposed methods. A patient monitoring block with 15 patients is considered. Each patient is assumed to be a WBAN with five sensor nodes and a body controller. The sensor nodes collect the body vital signs such as blood glucose, blood pressure, body temperature, ECG and EEG. The measured data are given to the body controller which is deployed on the human body. The patient monitoring system consists of 15 body controllers which form the tree structure for the proposed model. The body controller transfers the data to the fog node (central coordinator) using the proposed algorithms. The fog node assigns priority to the data and sends the prioritized data to the physician through the cloud server to meet the emergency situations. The data processing and computation are done within the fog node and only the consolidated report is sent to the physician through the cloud server. The local server in the proposed network is called here as the cloud server. The cloud server assigned here is mainly to connect to the external network. The fog node avoids congestion in the network, reduces the computation time by performing all operations in the fog node itself. It also minimizes the storage size and redundant data package (only important data is sent to the server), and decreases the time delay between the source and the destination.

The designed MCPS algorithm is used to transfer data towards the central coordinator. The developed fuzzy based dynamic time slot allocation is utilized to improve the reliability and network lifetime.

### 3.2. Block Diagram of a Fog-Based WBAN

**Figure 2:**
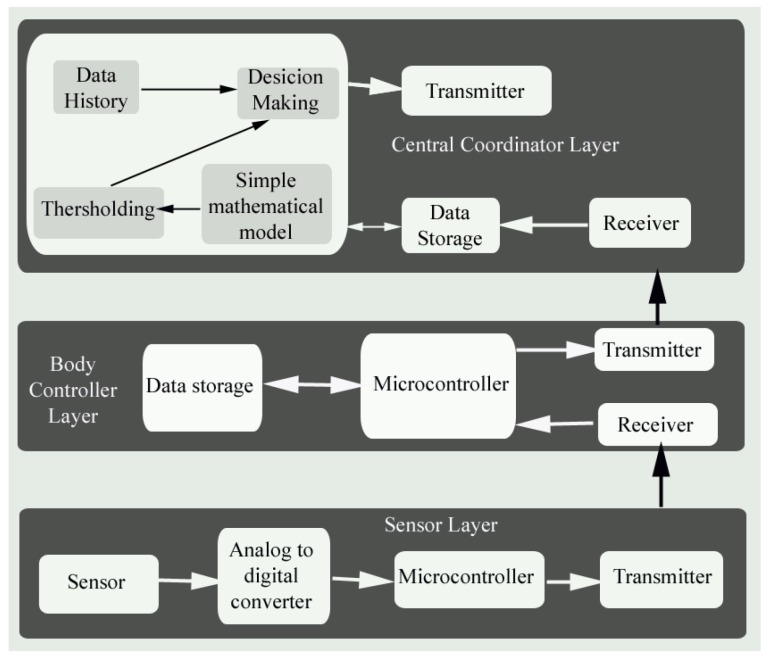
Block diagram of a fog-based WBAN.

The functional block diagram of the proposed fog-assisted architecture for the real-time health monitoring system is shown in Figure 2. The three layers in the monitoring framework are as follows:

1. Sensor layer
2. Body controller layer
3. Central coordinator layer

The sensor layer collects the body vitals and processes the signals that must be transmitted to the next layer. The body controller layer stores and transmits the data about the fog layer or central coordinator layer. Here, simple mathematical modeling has been used to make a decision regarding the priority of the data. From this layer, the prioritized data are transmitted to the physician through the cloud server. The role of a fog node in the proposed model are:

1. Collecting the human vital signs from sensor nodes
2. Computing and analyzing the sensed data using simple modeling techniques
3. Sending the consolidated report to the cloud server
4. Assigning the priority of the sensed data
5. Coordinating operations of the body sensor nodes

The patient vitals is transmitted to the base station through the body controllers, using a trusted tree formed with n number of body controllers within each block. The fog nodes determine the priority of the data with the help of the prioritization scheme and send the data towards the destination through a cloud server. The back-end part of the system is the cloud server, whose function includes storing, processing, and transmission of data along with back-end services for real-time data interpretation and visualization. The tree formation between the body controllers and the next hop node selection and dynamic time slot assignment is explained in the following sections.

## 4. Tree Formation and Cost Function Evaluation

### 4.1. Tree Formation

The first step in the initialization phase is the tree formation with the available set of sensor nodes and the central coordinator (CC) or the root node. Initially, the root node broadcasts the CC announcement to all the neighboring nodes. The CC announcement includes a sequence number, number of the visited devices, available energy, queue length, and all the necessary parameters to select the parent node. CC announcement is broadcast based on a sink timer. Initially, the one-hop neighboring nodes will receive the announcement from the root node. Based on the received sequence number and hop count, the tree is formed with selected parents and children. The detailed psedocode for the tree formation is given as follows:

1. The root node broadcasts a CC announcement using a sink timer
2. One-hop connected devices receive the message
3. If the received sequence number is new add the previous hop forwarder in the tentative parent list
4. If the received sequence number is not new but if its hop count is less than the previous one, then add it to the tentative parent list
5. Execute MCPS algorithm (Algorithm 1) to select the best parent node

### 4.2. Link Cost Function for Next-Hop Selection

The objective of the link cost function is to select a node with minimum link cost as the best parent node. It [40] is based on the parameters such as: residual energy, queue size, link reliability, the distance between the nodes and the available bandwidth. Consider the variable *x*, where x is given as:

(1) $$x=w_{1}\times \frac{e_{r}}{e_{i}}+w_{2}\times \frac{q_{a}}{q_{i}}+w_{3}\times R_{i,j}\left(n\right)+w_{4}\times \frac{d}{c}+w_{5}\times \frac{b_{r}}{b_{a}},$$

where, $e_{r}$, $e_{i}$, $q_{i}$ and $q_{a}$ are residual energy, initial energy, initial queue size, and current available queue size of node j respectively. $R_{ij}$(*n*) is the current round link reliability between the nodes *i* and *j*, which is estimated from Equation (3). The metrics *d*, *c*, $b_{a}$, and $b_{r}$ represent the distance between the two nodes, the coverage of a node, the required bandwidth, and the residual bandwidth respectively. $w_{1}$, $w_{2}$, $w_{3}$, $w_{4}$, and $w_{5}$ are the weighted coefficients, where

(2) $$\sum_{i=1}^{5}W_{i}=1$$

The link reliability between any two sensor nodes ($R_{ij}$) is estimated from the exponentially weighted moving average, which is given as follows:

(3) $$R_{i,j}\left(n\right)=\left(1-\gamma \right)\times Ri,j\left(n-1\right)+\gamma \times \frac{N_{t}}{\tau_{tr}},$$

where $N_{t}$ is the total number of successful packet transmission attempts through the link between the nodes *i* and *j*, *n* is the index number of the round, $\tau_{tr}$ is the total number of successful transmission and re-transmission attempts of all data packets, and γ is the average weighting factor.

The distance between the two nodes can be calculated using Equation (4):

(4) $$d^{2}={\left(x_{1}-x_{2}\right)}^{2}+{\left(y_{1}-y_{2}\right)}^{2},$$

where *x* and *y* are the coordinates.

The expression for the link cost function is expressed as:

(5) $$LC_{i,j}=e^{-x},$$

where, *x* is expressed in Equation (1). The range of link cost function is (0.367,1). The mentioned link cost considered five factors in order to enhance the QoS performance of the network. The energy metric aims to stabilize the energy between the nodes, queue size metric attempts to reduce the queuing delay, the link reliability improves the reliability of the network, node coverage and the distance between the nodes are used to decrease the number of re-transmission attempts, and the residual bandwidth increases the packet delivery ratio of the network by utilizing the available bandwidth resource of the network.

### 4.3. Minimum Cost Parent Selection Algorithm

To find the best parent node, the proposed minimum cost parent selection (MCPS) algorithm is used whenever a node receives an announcement from the neighboring nodes. According to this algorithm, the best parent node will be the one with a minimum hop, minimum cost, and the shortest distance from the child node. Since it utilizes the minimum link cost, minimum hop, and the shortest distance between nodes, it satisfies the required QoS for WBANs. The selection of best parent nodes from the tentative parent list is depicted in Algorithm 1.

**Algorithm 1** Best parent node selection algorithm.**Initialization:** $LC_{ij}$—link cost function between sensor nodes *i* and *j* $C_{m}$—maximum link cost = 1 $N_{id}$ = −1 $h_{n}$—highest number of hops nid—node Identifier of node *j* $C_{n}id$—link cost of node *j* NNi s1, s2, ….., sm—set of neighboring nodes of node *i*, 1≤i≤N,1≤m≤N BNHi—best parent node of $NN_{i}$ $N_{md}$—node with minimum distance from child node 1: **for** each node in the list $NN_{i}$
**do** 2: compute link cost: $LC_{ij}$ using Equation (9) 3: **end for** 4: **for** each node, *j*, in the list *NN_i_*
**do** 5: nid = node Id of *j* 6: **if**
$h_{n}$ > $h_{nid}$
**then** 7:  $C_{m}=C_{nid}$ 8:  $h_{n}=h_{nid}$ 9:  $N_{id}=nid$ 10: **else** 11:  **if**
$h_{n}==h_{nid}$
**then** 12:   **if**
$C_{m}$ > $C_{nid}$
**then** 13:    $C_{m}=C_{nid}$ 14:    $N_{id}=nid$ 15:   **else** 16:    **if**
$C_{m}=C_{nid}$
**then** 17:     $N_{id}=N_{md}$ 18:    **end if** 19:   **end if** 20:  **end if** 21: **end if** 22: $BNH_{i}=N_{id}$ 23: **end for**

## 5. Fuzzy-Based Dynamic Time Slot Allocation

Once the traffic is generated, the initial equal slot assignment may fail due to the dynamic conditions in the networks such as traffic flow, buffer availability, energy consumption by each node, and so on. Therefore, each of these parameters in the network is highly unpredictable. The solution proposed to this situation is the dynamic time slot allocation technique (DTS), where the slots are allocated to nodes depending on the packet interval, buffer availability, and the remaining energy of each node. In order to improve the reliability and efficiency of packet transmission, fuzzy-based dynamic slot allocation has been proposed. Fuzzy logic [41] can give an appropriate solution or can integrate many factors to solve an evaluation problem. In this method, it is used to find a dynamic time slot to each node based on the mentioned factors.

### 5.1. Fuzzification

**Figure 3:**
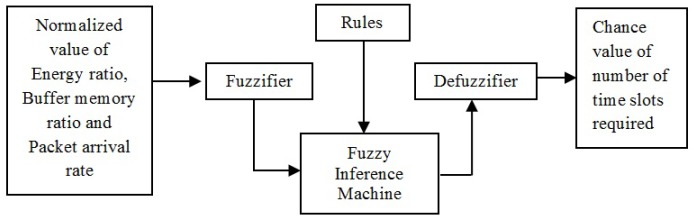
Block diagram of fuzzy model.

In the first step of fuzzification, the crisp inputs are converted into their corresponding linguistic values, which are represented through the use of fuzzy sets [42]. Each fuzzy set is related to a membership function that describes the way in which each crisp input is associated with the fuzzy set. The fuzzy model is shown in Figure 3. For the slot allocation to each node, the three input variables of fuzzy are energy ratio (ER), packet arrival rate (PAR), and buffer memory ratio. The fuzzy model uses three linguistic terms (low, medium, and high) in order to partition the input variable. To define each term, different membership functions such as Gaussian, S, and Z functions are used.

#### 5.1.1. Energy Ratio

The energy ratio (*ER*) variable indicates the ratio of available energy ($E_{r}$) to the initial energy ($E_{i}$) at each node and is given as follows:

(6) $$ER=\frac{E_{r}}{E_{i}}.$$

Equations (7)–(9) explain the partitioning of the variable energy ratio. Fuzzy rules for available energy and slot allocation are the following:

1. If ER is high, then slot allocated value is high.
2. If ER is medium, then slot allocated value is medium.
3. If ER is low, then slot allocated value is low.

(7) $$u_{l}\left(x\right)=\left\{\begin{array}{cc} 1 & x\in (0,0.3)\\ 1-2{\left(\frac{x-0.3}{0.2}\right)}^{2} & x\in (0.3,0.4)\\ 2{\left(\frac{x-0.5}{0.2}\right)}^{2} & x\in (0.4,0.5)\\ 0 & x>0.5 \end{array}\right.$$

(8) $$u_{m}\left(x\right)=\left\{\begin{array}{cc} e^{-}\left(\frac{{\left(x-c\right)}^{2}}{2\sigma^{2}}\right) & x\in (0.2,0.8)\\ 0 & \mathrm{otherwise} \end{array}\right.$$

(9) $$u_{h}\left(x\right)=\left\{\begin{array}{cc} 1 & x<0.5\\ 2{\left(\frac{x-0.5}{0.2}\right)}^{2} & x\in (0.5,0.6)\\ 1-2{\left(\frac{x-0.7}{0.2}\right)}^{2} & x\in (0.6,0.7)\\ 1 & x>0.7 \end{array}\right..$$

#### 5.1.2. Buffer Memory Ratio

The second input variable is the Buffer Memory Ratio (*BMR*), which can be calculated according to Equation (10),

(10) $$BMR=\frac{m_{a}}{m_{max}}$$

where $m_{a}$ is the available memory in a node and $m_{max}$ is the maximum memory allotted to that node. Equations (11)–(13) explain the partitioning of the variable BMR. Fuzzy rules for BMR and slot allocation are the following:

1. If BMR is high, then slot allocated value is high.
2. If BMR is medium, then slot allocated value is medium.
3. If BMR is low, then slot allocated value is low.

(11) $$u_{l}\left(x\right)=\left\{\begin{array}{cc} 1 & x\in (0,0.15)\\ 1-2{\left(\frac{x-0.15}{0.35}\right)}^{2} & x\in (0.15,0.325)\\ 2{\left(\frac{x-0.5}{0.3}\right)}^{2} & x\in (0.325,0.5)\\ 0 & x>0.5 \end{array}\right.$$

(12) $$u_{m}\left(x\right)=\left\{\begin{array}{cc} e^{-}\left(\frac{{\left(x-c\right)}^{2}}{2\sigma^{2}}\right) & x\in (0,1)\\ 0 & \mathrm{otherwise} \end{array}\right.$$

(13) $$u_{h}\left(x\right)=\left\{\begin{array}{cc} 1 & x<0.5\\ 2{\left(\frac{x-0.5}{0.3}\right)}^{2} & x\in (0.5,0.65)\\ 1-2{\left(\frac{x-0.8}{0.3}\right)}^{2} & x\in (0.65,0.8)\\ 1 & x\in (0.81) \end{array}\right.$$

#### 5.1.3. Packet Arrival Rate

The packet arrival rate (*PAR*) in the network is estimated with the help of the exponentially weighted moving average (EWMA) method. It is defined as follows:

(14) $$PAR=\alpha_{1}\times pr_{avg}+\left(1-\alpha_{1}\right)\times pr_{cur},$$

where $\alpha_{1}$ is the weighting factor that takes the value within the range from 0.1 to 0.9. $pr_{avg}$ is the average of the previously arrived packet rate, $pr_{cur}$ is the current packet arrival rate. Equations (15)–(17) explain the partitioning of the input variable *PAR*. Fuzzy rules for *PAR* and slot allocation are as follows:

1. If PAR is high, then the slot allocated value is high.
2. If PAR is medium, then the slot allocated value is medium.
3. If PAR is low, then the slot allocated value is low.

(15) $$u_{l}\left(x\right)=\left\{\begin{array}{cc} 1 & x\in (0,0.15)\\ 1-2{\left(\frac{x-0.15}{0.35}\right)}^{2} & x\in (0.15,0.325)\\ 2{\left(\frac{x-0.5}{0.3}\right)}^{2} & x\in (0.325,0.5)\\ 0 & x>0.5 \end{array}\right.$$

(16) $$u_{m}\left(x\right)=\left\{\begin{array}{cc} e^{-}\left(\frac{{\left(x-c\right)}^{2}}{2\sigma^{2}}\right) & x\in (0,1)\\ 0 & \mathrm{otherwise} \end{array}\right.$$

(17) $$u_{h}\left(x\right)=\left\{\begin{array}{cc} 1 & x<0.5\\ 2{\left(\frac{x-0.5}{0.3}\right)}^{2} & x\in (0.5,0.65)\\ 1-2{\left(\frac{x-0.8}{0.3}\right)}^{2} & x\in (0.65,0.8)\\ 1 & x\in (0.81) \end{array}\right..$$

The membership function for the output allocated slot value can be explained using Equations (19)–(23).

(18) $$u_{r}l\left(x\right)=\left\{\begin{array}{cc} 1 & x\in (0,0.1)\\ 1-2{\left(\frac{x-0.1}{0.2}\right)}^{2} & x\in (0.1,0.2)\\ 2{\left(\frac{x-0.3}{0.2}\right)}^{2} & x\in (0.2,0.3)\\ 0 & x>0.3 \end{array}\right.$$

(19) $$u_{l}\left(x\right)=\left\{\begin{array}{cc} 0 & x<0.1\\ e^{-}\left(\frac{{\left(x-c\right)}^{2}}{2\sigma^{2}}\right) & x\in (0.1,0.5)\\ 0 & x>0.5 \end{array}\right.$$

(20) $$u_{m}\left(x\right)=\left\{\begin{array}{cc} 0 & x<0.3\\ e^{-}\left(\frac{{\left(x-c\right)}^{2}}{2\sigma^{2}}\right) & x\in (0.3,0.7)\\ 0 & x>0.7 \end{array}\right.$$

(21) $$u_{h}\left(x\right)=\left\{\begin{array}{cc} 0 & x<0.5\\ e^{-}\left(\frac{{\left(x-c\right)}^{2}}{2\sigma^{2}}\right) & x\in (0.5,0.9)\\ 0 & x>0.9 \end{array}\right.$$

(22) $$u_{r}h\left(x\right)=\left\{\begin{array}{cc} 1 & x<0.7\\ 2{\left(\frac{x-0.7}{0.2}\right)}^{2} & x\in (0.7,0.8)\\ 1-2{\left(\frac{x-0.9}{0.2}\right)}^{2} & x\in (0.8,0.9)\\ 1 & x>0.9 \end{array}\right..$$

Table 1 defines the fuzzy inference rules for the selection of the optimal slot value for each node. The fuzzy rules consist of a series of conditional statements of “if–then” type. The rating is given as “low”, “rather low”, “medium”, “rather high”, and “high”. If the normalized input variables for ER, BMR, and PAR is all low, then the chance value for the required number of slots for the particular node is expected to be low. Similarly, if the normalized input variables are all high, then the chance value for the required number of slots for a certain node is expected to be low. The remaining chances occur between these two extremes. The inference system used to find the chance value fuzzy variable is the Mamdani fuzzy inference system. The results of all fuzzy rules are in fuzzy values and are converted into crisp values based on the centroid of area $U_{CoA}$:

(23) $$U_{COA}=\frac{\sum_{x=1}^{n_{r}}u_{x}Z\left(u_{x}\right)}{\sum_{x=1}^{n_{r}}Z\left(u_{x}\right)},$$

where Z(u−x) is the membership function of all aggregated outputs, $u_{x}$ is the centroid of the area, and $n_{r}$ is the number of fuzzy rules.

### 5.2. Comparison of Time Slot Allocation

**Figure 4:**
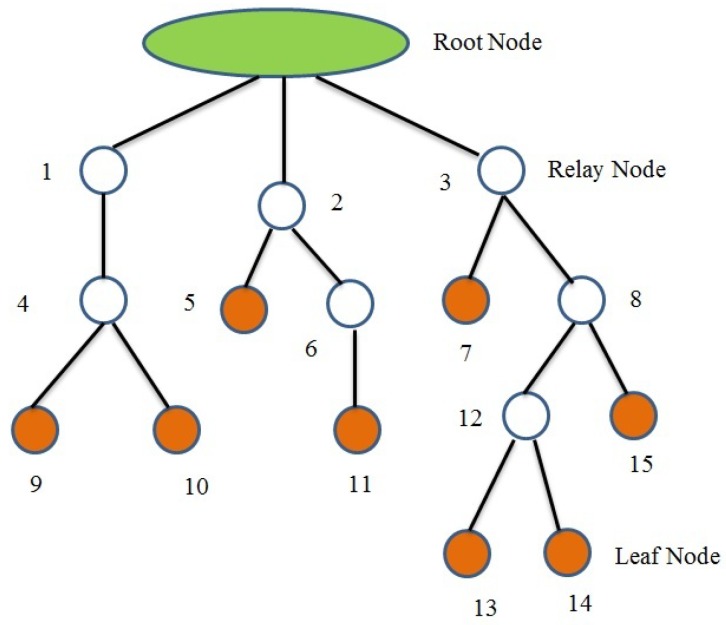
Example of a tree network.

**Figure 5:**
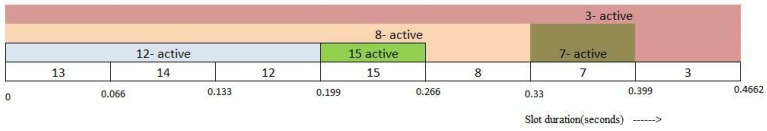
Slot allocation and parent node active state duration in conventional method.

**Figure 6:**
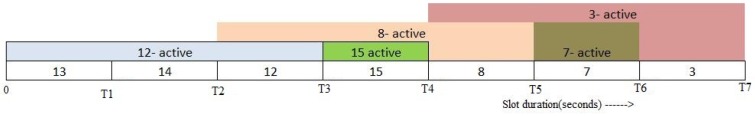
Slot allocation and parent node active state duration in DTS method.

Consider the tree structure shown in Figure 4. The tree structure has 15 nodes and a root node. The number of nodes are selected in random manner. The root node has three direct children node 1, 2, and 3. The whole tree can be divided into three branches. The branch I include nodes 1, 4, 9, and 10. Branch II has nodes 2, 5, 6, and 11. Nodes 3, 7, 8, 12, 15, 13, and 14 constitute branch III. Any node in the tree can be a child node, a relay node, or a leaf node (which does not have any child). For example, node 3 has direct children 7 and 8; the leaf nodes are 7, 13, 14, and 15; the relay nodes are 3, 8, and 12. Assume that the total transmission time for all nodes is 1 s. With a total of 15 nodes, the equal slot duration for each node is 0.0666 s (1/15). Hence, the total slot duration for nodes 1, 2, and 3 are 0.266 s, 0.266 s, and 0.466 s respectively. This equal slot allocation is used for the conventional sensor networks. The proposed DTS method uses dynamic slot allocation to enhance network performance. The slot allocation to each node depends on the relay nodes, child nodes, and the leaf nodes. In the conventional method of equal slot allocation, each parent node must be active during the entire time duration of its child and leaf nodes. This leads to higher energy consumption. Hence, the dynamic slot allocation method is adopted in the DTS scheme such that the parent needs to be active only during its own and direct child slot duration. This is represented in Figure 5 and Figure 6. It shows the comparison between the two methods with respect to the duration of an active state for parent nodes for the branch III. The branch has nodes 3, 7, 8, 12, 13, 14, and 15. According to the conventional method, the node 3 has to be active for a duration of 0.4662 s and node 8 must remain active for a duration of 0.333 s. The DTS method reduces the active duration of nodes 3 and 8 to 0.1998 s which also results in reduced energy consumption.

## 6. Performance Analysis and Comparison

### 6.1. Simulation Setup

The performance was evaluated using the network simulator version 2 (NS-2) simulating tool. NS-2 is an object-oriented discrete event simulator for research in wired and wireless networks that can simulate newly designed network protocols. It has a number of wireless networking supported platforms and protocols for detailed study of simulated results. A random WBAN network was considered with 15 sensor nodes. The network used IEEE 802.15.4 as the MAC protocol. The simulation time was selected as 200 s, and the packet interval was varied from 0.1 s to 3 s in steps of 0.5. Table 2 summarizes the simulation parameters used.

### 6.2. Performance Metrics and Results

The performance of the proposed technique was validated with the help of key factors such as packet delivery ratio (PDR), average end to end delay, and average energy consumption. The experiments were conducted for two sets, based on the selected simulation time and the packet interval time. The proposed DTS mechanism was compared with the basic IEEE 802.15.4 standard and the TMP protocol [24].

**Figure 7:**
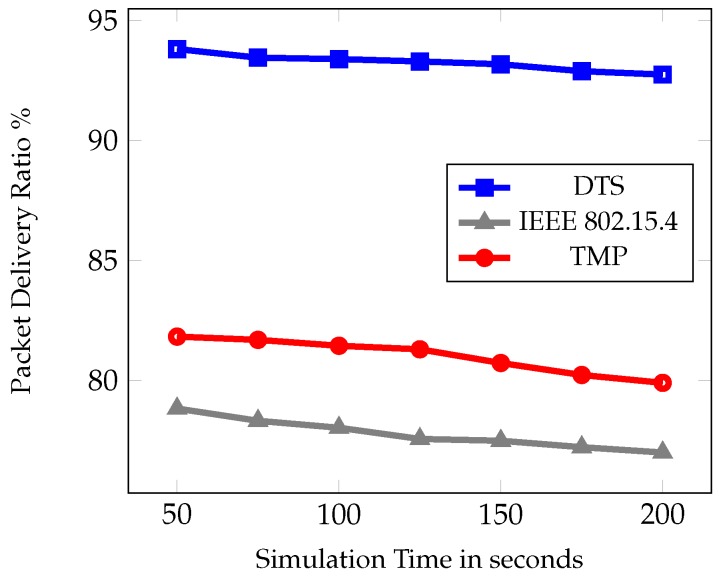
Packet delivery ratio under different simulation times.

**Figure 8:**
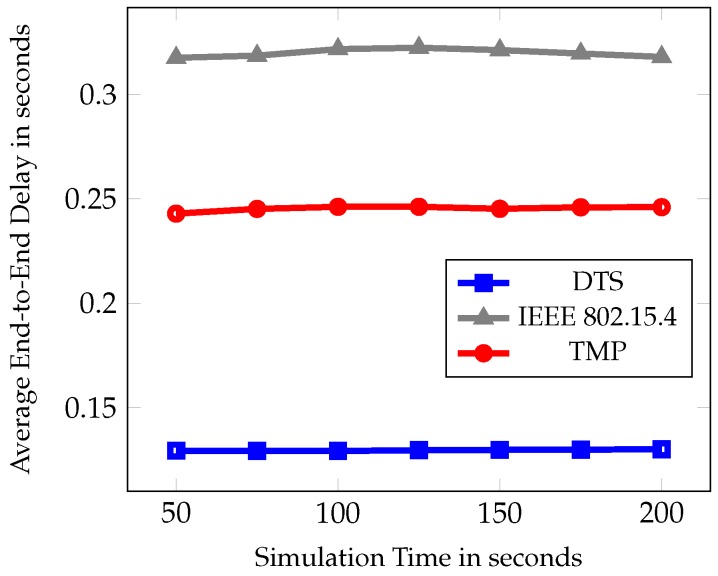
Average End-to-End Delay under different Simulation Time.

**Figure 9:**
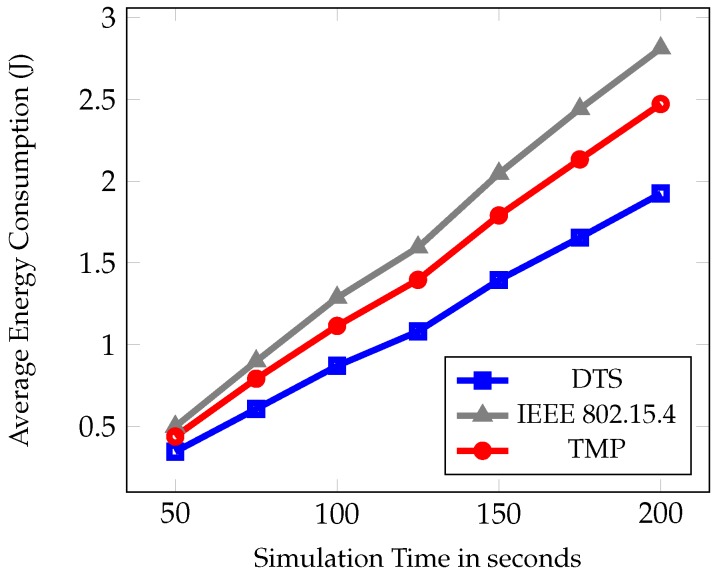
Average energy consumption under different simulation time.

In the first set of experiment, the simulation time was varied as 50, 75, 100, 125, 150, 175, and 200 s. Figure 7, Figure 8 and Figure 9 show the comparative results of PDR, average end to end delay, and average energy consumption of IEEE 802.15.4, TMP and the proposed DTS protocols.

Figure 7 depicts the PDR from a source node to the root node. It was measured as the percentage of the total number of successfully received packets at the root node to the number of packets transmitted from the source node. The figure shows that PDR was highest for DTS method. This is due to the link reliability function used in the best next hop selection algorithm. It ensured the best path between the source node and the root node by reducing the packet loss. On the other hand, the TMP protocol mainly concentrated on the time slot allocation to minimize the slot wastage. In IEEE 802.15.4 standard the data transmission was based on the CAP and CFP transmission. In comparison, DTS outperformed the TMP and IEEE 802.15.4 standard by 12% and 15% respectively.

In Figure 8, the average end-to-end delay is shown with varied simulation time. It is the average time taken by the packet to reach the root node from the source node. DTS scheme dynamically allocated the time slot based on the available energy, buffer memory, and packet arrival rate. It allocated the slot dynamically. Hence the slot wastage gets reduced where unnecessary waiting time is minimized for those nodes in the queue. In TMP, computational methods were utilized for MAC parameter tuning and duty cycle adjustment which contributes lesser than the DTS method. There was a 47% and 59% reduction in average end-to-end delay when compared to TMP and IEEE 802.15.4 MAC Standard.

The average energy consumption is depicted in Figure 9, where the DTS scheme has the lowest energy consumption. The MCPS algorithm proposed for next hop selection is based on the available energy in each node. The dynamic time slot allocation based on fuzzy rules used the energy ratio to utilize the available energy resources effectively. In TMP, the energy ratio consideration is less when compared to DTS. The percentage of reduction in average energy consumption of DTS is 22% and 31% respectively.

**Figure 10:**
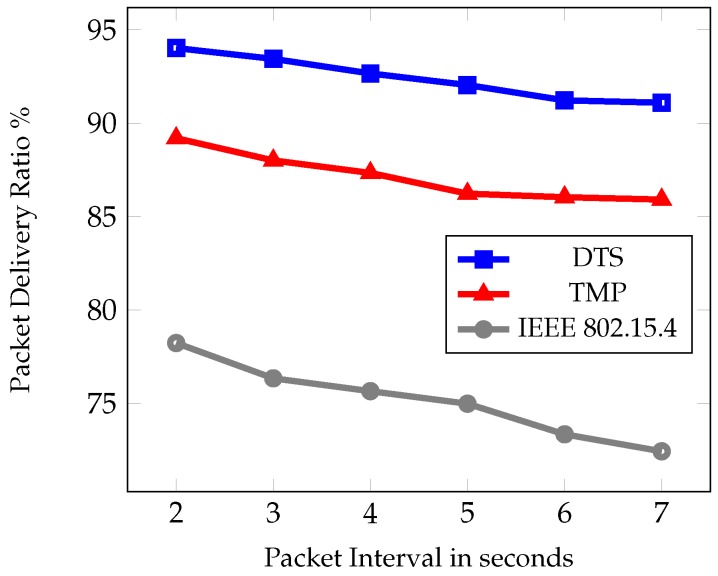
Packet delivery ratio under different packet intervals.

**Figure 11:**
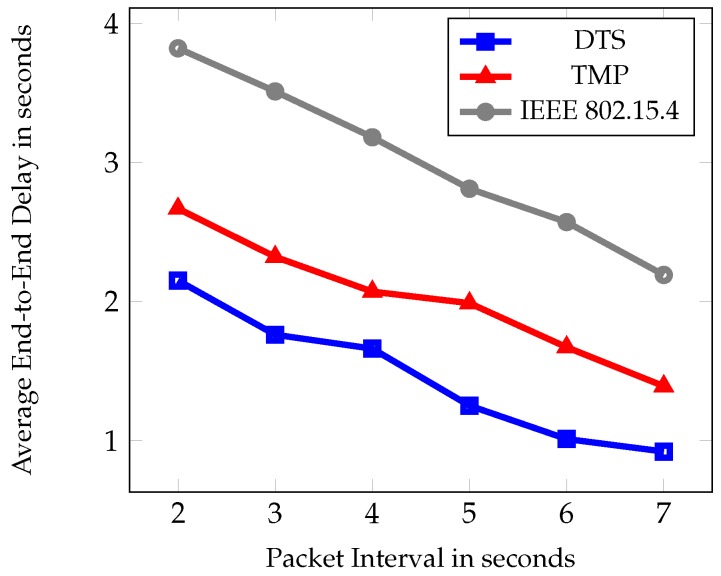
Average end-to-end delay under different packet intervals.

**Figure 12:**
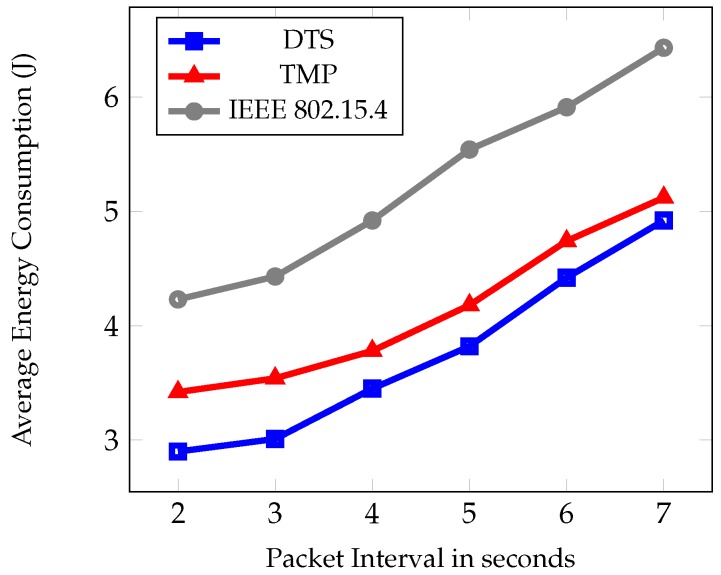
Average energy consumption under different packet intervals.

The second set of experiments was based on different packet interval time such as 2–7 s. Figure 10, Figure 11 and Figure 12 show the comparative results of PDR, average end to end delay, and average energy consumption of IEEE 802.15.4, TMP and the proposed DTS protocols. Figure 10 shows the PDR for different packet interval. As packet interval decreases the traffic load increases hence there is a decrease in PDR. This is due to the less number of packets generated during low traffic conditions. As traffic load increased, more packets were injected into the networks and there was an increase in the PDR. As packet interval increased there was a decrease in the PDR. This is due to the congestion and collisions in low traffic load. The data packets could reach the root node easily at high packet intervals. The DTS performed better than the TMP and IEEE 802.15.4 standard in terms of PDR by 5% and 17% respectively.

It is obvious from the figure that as packet interval increases the average end-to-end delay decreases. The traffic will be less at longer packet interval and at the shorter interval the traffic will be heavy. More packets were injected into the network during high traffic resulting in network congestion and reduced buffer size. Therefore, packets cannot reach the root node easily resulting in increased end-to-end delay. During high packet interval, the traffic load decreased and the packets can reach the root node easily. Hence, the average end-to-end delay decreased. DTS allocated the time slot by considering the available buffer memory in each node. Therefore, the dynamic time slot selection reduced the average end-to-end delay in the network. The percentage of reduction when compared with TMP and IEEE 802.15.4 was 41% and 43% respectively.

Figure 12 shows that as the packet interval increased the energy consumption also increases. This is due to the decrease in the traffic load with an increase in the packet interval. An increase in the packet interval increased the idle listening time and the time required to transmit control overheads. This resulted in an increase in energy consumption. If the packet interval was at minimum, the listening time and the control overhead transmission time is also minimum. Hence, the energy consumption was minimum. In addition to this, ER in fuzzy rules and the remaining energy in the link cost function helped control the rise in energy consumption when compared to the other protocols. Figure 11 shows that the DTS scheme had the lowest energy consumption than the existing protocols. The percentage of decrease was 25% and 39% when compared with TMP and IEEE802.15.4 standard.

From the two sets of simulation results, it is obvious that there is a considerable improvement in packet delivery ratio with respect to the compared protocols. Hence, the dropping ratio was high, thereby resulting in a better packet delivery ratio. Similarly, the average delay and the energy consumption was also reduced considerably due to the energy efficient link cost function used in the routing layer and the energy ratio considered in the time slot allocation method.

## 7. Conclusions

The major challenges identified in real-time patient monitoring WBANs are the higher response time, lower reliability, and higher energy consumption. These shortcomings can be addressed in MAC layer using dynamic time slot allocation instead of fixed slot allocations. In this paper, a fog-assisted network is utilized for a real-time patient monitoring setup. The fog layer (central coordinator) is deployed at the edge of the network to reduce the response time and transmission errors. This makes it suitable for emergency medical applications which carries bursty data. An energy-efficient, cost-based objective function and an MCPS algorithm is designed for routing the data packets to the coordinator node. A new dynamic time slot allocation method called DTS has been proposed for allocating dynamic slots to the sensor nodes. It minimizes the unnecessary slot wastage and waiting time of packets in the queue. The slot allocation is designed based on the fuzzy logic with input variables as energy ratio, buffer memory ratio, and packet arrival rate. The chance value for the number of slots allocated is determined with the help of fuzzy inference rules. The results reveal that the DTS is capable of achieving a relevant enhancement in packet delivery ratio (12% and 15%), a significant reduction in average end to end delay (47% and 59%) and average energy consumption (22% and 31%) in comparison with TMP and IEEE 802.15.4 respectively. Future work will include the enhanced version of the proposed model for a specific disease prediction which is based on different data rate patient vitals. Also, the fog-assisted network can be made more secure by implementing new data encryption and authentication methods.

## Figures and Tables

**Table 1:** Fuzzy inference rules.

ER	BMR	PAR	Required Time Slots
Low	High	Low	Rather low
Low	High	Medium	Medium
Low	High	High	Rather high
Low	Medium	Low	Low
Low	Medium	Medium	Rather low
Low	Medium	High	Medium
Low	Low	Low	Low
Low	Low	Medium	Low
Low	Low	High	Rather low
Medium	High	Low	Medium
Medium	High	Medium	Rather high
Medium	High	High	High
Medium	Medium	Low	Rather low
Medium	Medium	Medium	Medium
Medium	Medium	High	Rather high
Medium	Low	Low	Low
Medium	Low	Medium	Rather low
Medium	Low	High	Medium
High	High	Low	Rather high
High	High	Medium	High
High	High	High	High
High	Medium	Low	Medium
High	Medium	Medium	Rather high
High	Medium	High	High
High	Low	Low	Rather low
High	Low	Medium	Medium
High	Low	High	Rather high

**Table 2:** Simulation parameters.

Simulation Parameters	Values
Number of WBANs	15
Number of sensors per WBAN	5
Frequency	2.4 GHz
Data rate	20–250 kbps
Simulation time	200 s
Data packet size	50–150 bytes
Control packet size	15 bytes
Superframe length	100 ms
Initial energy of sensor nodes	100 J
Energy consumption: Transmission	16.7 nJ
Energy consumption: Reception	36.1 nJ
Energy consumption: Amplifier	1.97 nJ

## Abbreviations

The following abbreviations are used in this manuscript:

WBAN	wireless body area network
DTN	delay tolerant network
CBSNs	collaborative body sensor networks
BSNs	body sensor networks
C-SPINE	collaborative-signal processing in node environment
SPINE	signal processing in node environment
DTN	delay tolerant network
FCFS	first-come-first-served
GTS	guaranteed time slot
ART-GAS	adaptive and real-time GTS allocation scheme
RPL	routing protocol for low-power and lossy
DODAGs	destination-oriented directed acyclic graphs
VELCT	velocity energy-efficient and link-aware cluster-tree
CAP	contention access period
CFP	contention free period
DCT	data collection tree
TBRP	tree-based routing protocol
QL-MAC	Q-learning medium access control
DT-SCS	decentralized time-synchronized channel swapping
TSCH	time-synchronized channel hopping
IoT	internet of things
IEEE	Institute of Electrical and Electronics Engineers
PAN	personal area network
WSN	wireless sensor network
BS	base station
CC	central coordinator
EEG	electroencephalogram
ECG	electrocardiogram
MCPS	minimum cost parent selection
DTS	dynamic time slot
MAC	medium access control
TDMA	time division multiple access
CSMA/CA	carrier sense multiple access with collision avoidance
QoS	quality of service
PHY	physical
TMP	tele-medicine protocol
ER	energy ratio
BMR	buffer memory ratio
PAR	packet arrival rate
CoA	centroid of area
NS-2	network simulator-2
GTS	guaranteed time slot
PDR	packet delivery ratio
